# Perspective from an NGO

**DOI:** 10.4103/0019-5545.43054

**Published:** 2005

**Authors:** R. Thara

**Affiliations:** Director, Schizophrenia Research Foundation (SCARF), Chennai

This commentary will largely deal with two main issues. One is based on the experiences at the Schizophrenia Research Foundation (SCARF) over the past 20 years. The second will deal with the costs and cost-effectiveness of psychosocial rehabilitation, an integral and important part of schizophrenia care.

## SCARF STUDY

The Schizophrenia Research Foundation is an NGO based in Chennai committed to schizophrenia care and research. SCARF runs an outpatient department and three residential facilities with a total inpatient strength of 150. Being a referral rehabilitation agency, most of the patients referred to us are chronic, treatment-re-fractory and moderate/severely disabled. Between 2000 and 2001, we included 68 persons with schizophrenia in a study to assess the efficacy of clozapine. As part of this yet unpublished study, the costs of treatment for a period of 6 months before and after starting clozapine was computed and a cost analysis done. This was, of course, based only on the cost of medication and blood tests, and did not include indirect costs, which remained the same for each patient.^1^

Of the 68 patients, 42 were males, and the mean duration of illness was around 12 years. The mean age of the sample was 37 years. Before they came to SCARF, most of them had been treated by multiple practitioners with almost all available neuroleptics in the Indian market and seemed to be responding poorly to them. They were all shifted to clozapine after the required blood tests were done.

After a period of 6 months, when we computed the costs before and after clozapine, we found to our surprise that despite the costs of blood tests, the total cost of treatment with clozapine had come down by nearly 25%. In all but 2 cases, the families were spending less on treatment. None of these patients had to be hospitalized and all of them continued as outpatients.

On delving further into the data, we concluded that the following factors might be responsible for this:

1. Before they were started on clozapine, most of these patients had been on a combination of at least 2–3 neuroleptics as well as some adjuvant drugs. These ranged from haloperidol and chlorpromazine to risperidone and olanzapine. These naturally led to increased costs.
2. When they were shifted to clozapine, in most cases, it was a single drug that was being used and the average dosage was around 200 mg per day. No adjuvants were required.
3. Almost all patients were on a single night-time dosage which improved compliance.

Despite some limitation in design, this study provided us with insights into the costs of schizophrenia in the Indian setting. This was also a cost-effectiveness study that in its simplest form compared the value of alternative treatments for the same patients.

Cost-effectiveness studies done on newer antipsychotics have shown that their effects relate to relapses, rehospitalization and hospital utilization.^2^,^3^

It appears that in the Indian setting, polypharmacy is a significant factor in increasing costs. It would be a worthwhile research exercise, even on a multicentre level, to compare not just the costs, but also the cost-effectiveness of treatment with conventional and newer antipsychotics. This would certainly provide guidelines for the kind of drugs that should be made available in various kinds of treatment settings, be they primary or tertiary care.

## PSYCHOSOCIAL INTERVENTIONS

It is now universally accepted that psychosocial interventions are an integral part of the comprehensive management of schizophrenia. However, there are very few studies in the world that have included these in the cost or cost-effectiveness studies of schizophrenia.

During recent years, there has been an increasing interest in studying the costs and cost-effectiveness of psychiatric services.^4^,^5^ This has also brought into focus service use measurement issues^6^ and stressed the importance of incorporating service use in outcome studies. Cost-effectiveness studies have an obvious reference to the possibilities of comparing the outcome of different services in varied settings. Most studies have measured the cost-effectiveness of specific intervention programmes on targeted groups of patients, but there are not many on a system level. This is also important from the policy angle, as it determines resource allocation.

It is important at this juncture to appreciate the differences between estimation of costs and cost-effectiveness. The costs of an illness include direct costs of treatment, medication, other interventions and travel to treatment centres. Indirect costs are less tangible and include time lost by the patient and caregiver, loss of productivity and burden on caregivers.

Treatment costs for illnesses such as schizophrenia have been estimated in several studies. In a review of the literature, Lindstrom^7^ and De Hert^8^ noted that most studies apply a prevalence-based approach in which costs are included only for resources used and losses incurred during the assessment period, of usually one year. It is the services used that are evaluated and not individual patients. The former estimates are often derived from national figures of health expenditure.^7^,^8^

Cost-effectiveness studies attempt to determine the ratio of costs to outcomes of a particular intervention or treatment, and to compare a standard intervention with an alternative one to determine if the alternative is more cost-effective. The goal is to establish priorities for the allocation of resources and to decide among alternative interventions for the same medical condition.

By contrast, cost-effectiveness studies do evaluate real patients, but many of them are in specific research conditions. Literature on cost-effectiveness studies for rehabilitation is sparse and there is little from the developing world. They are probably more difficult and challenging to carry out for the following reasons:

1. The process of rehabilitation itself varies greatly from one setting to the other and does not always include identical components. There is also this dispute as to what actually constitutes rehabilitation. For example, are they facility-based such as outpatient, day care, institutional setting or community care? On the other hand, are they activity-based such as vocational training, social skills and assertive training, education, etc? In almost all developing countries and in many developed ones, the family also plays a critical role in rehabilitation and one cannot discount this while assessing cost-effectiveness.
2. Parameters of outcome: This is relatively straightforward in interventions involving medications alone and are generally clinical improvement, severity of side-effects and reduction/prevention of relapses. Both the intervention (dosage of medication) and the outcomes are well-defined and easy to measure. This, however, is not the case with rehabilitation. The outcomes are multiple and complex and may be so closely intertwined with each other that it could be difficult to tease out the individual elements. These could be:
  - reduction of social disability
  - increase in adaptive functioning (self-care and social skills)
  - decrease in the patient's psychotic and bizarre behaviour
  - stabilization of the dosage of medication required
  - reduction of recidivism
  - maintenance in the community
  - reduction of family burden
  - enhanced life satisfaction and quality of life, etc.
  - employability
  Apportioning weightage to each of these to measure cost--effectiveness is therefore a challenging task.
3. Factors such as health insurance and social security could also impact the process, but these are neither consistent nor uniform throughout the country.
4. Direct costs are easier to measure than indirect costs which, however, are an integral part of rehabilitation. For instance, a Belgian study did address the issue of unemployment.^8^
5. In several countries, much of the burden of care has been transferred from services to patients and families.^9^ Although the shift may imply direct cost savings for the government,^10^ it results in higher direct and indirect costs to the government. This shift in burden does not necessarily result in reduction of costs.

On account of the above-mentioned factors, measuring cost-effectiveness in a rehabilitation setting could be intriguing and complex, and hence attempts to do this have probably been sparse.

Paul and Lentz^11^ conducted a detailed cost-effectiveness analysis of the comparative dollars spent and saved by different treatment programmes over a 6-year period. Although staff: patient ratios were equal between psychosocial programmes and the hospital-based one, staffing costs were higher for the psychosocial programmes. The greater efficiency and effectiveness of the psychosocial programmes in producing successful discharge and community tenure resulted in considerable economic gain since it was found that aftercare is much less expensive than continued hospitalization. Purely on the basis of economic factors, both the psychosocial programmes were more effective than traditional hospital treatment, and the social learning programme emerged as the treatment of choice.^11^

## PSYCHOSOCIAL REHABILITATION (PSR) IN DEVELOPING COUNTRIES

Most developing countries are grappling with very low budgetary and resource allocation for health as a whole, of which mental health is a small part. Rehabilitation being no fancy subject attracts donors and is of low priority for state funding. Almost all rehabilitation activities are carried out in non-governmental settings by voluntary agencies, which also face the ongoing struggle of resource mobilization. Families look after almost all patients. There is no insurance coverage for mental disorders and no social security system in place. Even professionals working in mental health rehabilitation settings are few and are always looking for greener pastures.

The rehabilitation programmes themselves are not standardized, and their content, duration and delivery are often dependent on the quality and quantity of the rehabilitation personnel available. Very often these programmes are client--specific and few models have been developed which can be generalized. There is practically no PSR in rural areas. In the study undertaken by SCARF in the rural areas of Thiruporur, we found that the most suitable elements of the rehabilitation programme were empowering the families and offering simple, culture-specific interventions such as distribution of livestock or fishing nets.^12^

It is in this scenario that the cost-effectiveness of PSR needs to be estimated, which is no mean task. It is, however, essential to undertake such an exercise to mobilize more resources for rehabilitation, not only from the respective countries but from donor agencies as well.

## IMPLICATIONS

Clinical guidelines, defined as ‘systematically developed statements to assist both practitioner and patient decisions in specific circumstances’, have become an increasingly familiar part of clinical care.

There has been no widely accepted successful way of incorporating economic considerations into guidelines. Unlike other areas of guideline development, there is little practical or theoretical experience to direct the incorporation of cost issues within clinical guidelines. However, the reasons for considering costs are clearly stated: ‘Health interventions are not free, people are not infinitely rich, and the budgets of [health care] programmes are limited. For every dollar's worth of health care that is consumed, a dollar will be paid. While these payments can be laundered, disguised or hidden, they will not go away’.^13^

In the USA it has been recommended that every set of clinical guidelines should include information on the cost implications of alternative preventive, diagnostic, and management strategies for each clinical situation. The stated rationale was that this information would help potential users to evaluate better the potential consequences of different practices.

However, the impending impact of the Trade-Related Aspects of Intellectual Property Rights (TRIPS) agreement, with the strict enforcement of patent laws, will almost certainly lead to a rise in drug costs in the coming years. This may influence the choice and cost-effectiveness of various drugs. The implications of these cross-cultural variations for policy and practice are the need to ensure a reliable supply of affordable psychotropic drugs in developing countries, trained healthcare professionals to use these drugs rationally, a concerted advocacy campaign to exclude drugs for severe psychiatric disorders from patent protection, and the development of psychosocial programmes to improve global outcomes.
